# Supplementary material for: Sense of agency at a temporally-delayed gaze-contingent display
Source: PLoS One. 2024 Sep 6;19(9):e0309998. doi: 10.1371/journal.pone.0309998 (PMC12139658; doi:10.1371/journal.pone.0309998)
Supplement: S1 File — (DOCX) [file pone.0309998.s001.docx]

Appendices

Appendix S1.

Table S1. List of Chinese characters used as stimuli. Each image contained nine characters with an identical number of strokes.

| Number of strokes | Chinese Characters |
| --- | --- |
| 15 | 魴 魪 䰻 魥 魭 魧 魦 魣 魳 魫 魶 魹 魷 魨 魸 䰽 魬 魮 魵 䰷 魲 |
| 16 | 鮎 鮒 鮃 鮓 鮑 鮗 鮖 䱀 魼 鮔 䱁 鮏 鮀 鮐 鮊 鮁 鮍 魾 鮇 鮅 鮄 鮋 䱂 魿 䱈 鮌 鮉 䱇 |
| 17 | 鮮 鮪 鮭 鮫 鮟 鮨 鮠 鮴 鮣 鮱 鮰 鮚 鮬 鮯 䱎 䱍 鮥 鮞 鮛 鮡 鮧 鮦 鮩 鮲 |
| 18 | 鯉 鯀 䱔 鯒 鯑 鯏 鮹 鯆 鯎 鯇 鯁 鯋 䱒 鮻 䱌 鮼 鯐 鮾 鮵 鮿 鮷 䱐 鮸 |
| 19 | 鯨 鯖 鯵 鯛 鯣 鯢 鯤 鯲 鯡 鯔 鯱 鯰 鯘 䱤 鯕 䱡 䱟 鯝 䱜 鯞 䱙 鯧 鯩 鯜 鯳 鯯 鰙 鯫 鯮 鯟 鯥 鯪 |
| 20 | 鰐 鰍 鰓 鰉 鰔 鰕 鰌 鰈 鰆 鰒 鰊 鰄 鰛 鯹 䱭 鰀 鰋 鰅 鯸 鯶 鰇 鯺 鯽 鯼 鰂 鰖 鯷 䱱 鰚 鰏 鯾 鯿 鰘 鰑 鰦 |
| 21 | 鰯 鰭 鰮 鰥 鰤 鰰 鰡 䱽 鰞 鰪 䱵 䱻 鰜 鰬 鰝 䱹 鰣 鰠 鰧 鰨 鰢 鰟 鰩 鰫 |
| 22 | 鱈 鰺 鰻 鱆 鱇 鰾 鰱 䲁 鰳 䲅 鰶 鰼 鰽 鱃 鱂 鰷 鰿 鱄 䲃 鱁 鱅 |
| 23 | 鰹 鱒 鱚 鱉 鱊 鱛 鱎 鱖 鱑 鱏 鱘 鱓 鱔 鱍 鱕 鱝 |
| 24 | 鱗 鱠 鱧 鱫 鱜 鱞 鱤 鱥 鱪 鱐 鱢 鱣 鱩 鱯 鱮 |

Appendix S2.

Task error rate for each participant, condition, and target. The error rates were often highest in the playback condition (Table S2). Participants often could not inspect all eight surrounding characters in the playback condition because the recorded gaze behavior often did not cover the entire character array.

Table S2. The task error rate for each participant, each condition, and target existence.

| Participant | | Delayed window condition | | | Playback condition | | | No window condition | | |
| --- | --- | --- | --- | --- | --- | --- | --- | --- | --- | --- |
|  |  | Trial counts | Error trial counts | Error rate | Trial counts | Error trial counts | Error rate | Trial counts | Error trial counts | Error rate |
| 1 | no-target | 461 | 7 | 1.5% | 29 | 1 | 3.4% | 24 | 1 | 4.2% |
|  | target | 432 | 17 | 3.9% | 31 | 6 | 19.4% | 12 | 2 | 16.7% |
|  | Total | 893 | 24 | 2.7% | 60 | 7 | 11.7% | 36 | 3 | 8.3% |
| 2 | no-target | 457 | 1 | 0.2% | 34 | 0 | 0.0% | 24 | 0 | 0.0% |
|  | target | 442 | 2 | 0.5% | 26 | 1 | 3.8% | 12 | 1 | 8.3% |
|  | Total | 899 | 3 | 0.3% | 60 | 1 | 1.7% | 36 | 1 | 2.8% |
| 3 | no-target | 457 | 3 | 0.7% | 35 | 0 | 0.0% | 24 | 0 | 0.0% |
|  | target | 443 | 15 | 3.4% | 25 | 1 | 4.0% | 12 | 0 | 0.0% |
|  | Total | 900 | 18 | 2.0% | 60 | 1 | 1.7% | 36 | 0 | 0.0% |
| 4 | no-target | 427 | 0 | 0.0% | 33 | 0 | 0.0% | 18 | 1 | 5.6% |
|  | target | 450 | 9 | 2.0% | 27 | 1 | 3.7% | 18 | 0 | 0.0% |
|  | Total | 877 | 9 | 1.0% | 60 | 1 | 1.7% | 36 | 1 | 2.8% |
| 5 | no-target | 459 | 0 | 0.0% | 32 | 0 | 0.0% | 24 | 0 | 0.0% |
|  | target | 440 | 3 | 0.7% | 28 | 9 | 32.1% | 12 | 0 | 0.0% |
|  | Total | 899 | 3 | 0.3% | 60 | 9 | 15.0% | 36 | 0 | 0.0% |
| 6 | no-target | 453 | 1 | 0.2% | 32 | 0 | 0.0% | 24 | 0 | 0.0% |
|  | target | 447 | 10 | 2.2% | 28 | 5 | 17.9% | 12 | 0 | 0.0% |
|  | Total | 900 | 11 | 1.2% | 60 | 5 | 8.3% | 36 | 0 | 0.0% |
| 7 | no-target | 453 | 4 | 0.9% | 32 | 3 | 9.4% | 24 | 0 | 0.0% |
|  | target | 437 | 32 | 7.3% | 28 | 15 | 53.6% | 12 | 0 | 0.0% |
|  | Total | 890 | 36 | 4.0% | 60 | 18 | 30.0% | 36 | 0 | 0.0% |
| 8 | no-target | 456 | 1 | 0.2% | 36 | 0 | 0.0% | 22 | 0 | 0.0% |
|  | target | 444 | 3 | 0.7% | 24 | 6 | 25.0% | 14 | 0 | 0.0% |
|  | Total | 900 | 4 | 0.4% | 60 | 6 | 10.0% | 36 | 0 | 0.0% |

Appendix S3.

We believe that the combinations of English letters could have similarly prevented attentional bias and shared similar spatial frequencies. Using the pools of English letters (Table S3) and Chinese characters (Appendix S1) to determine which language to use, a pilot experiment was undertaken to establish whether Chinese characters or English letters would be more appropriate. Visual search tasks were conducted with a gaze-contingent window under two conditions: (1) stimuli were composed of Chinese characters (Fig 1) or English letters (Fig S1), and (2) the window was presented with a temporal delay relative to the participant’s gaze position using five different temporal delays (which were set at 100 ms intervals from 0 ms to 500 ms). Each trial was performed 30 times for each participant. The results of the pilot study revealed that the average response time for the visual search task with English letters was approximately 2 seconds shorter than that for Chinese characters (Fig S2). As the initial intention of this pilot test was to find a stimulus set that would enable the participants to control the gaze-contingent for an adequate time to collect eye movement data, the aforementioned result implied that Chinese characters were more suitable for this experimental design. Therefore, as shown in Appendix S1, 239 Chinese characters were used to generate stimulus images for the experiment.

Table S3. List of letter pairs used for the stimuli of pilot experiments using Courier New font. Four pairs were selected due to their similar appearance. One pair was selected randomly from the four. Stimuli and mask images were generated from the pairs. Stimuli images contained one target character and eight different uniform background characters (e.g., “b” for the target character, “p” for the background character). Mask images contained nine uniform characters (i.e., “b” or “p”). Two stimuli and two mask images were created from one pair. Using 2 × 2 combinations, four combinations were created from one pair (16 combinations in total).

| Letters |
| --- |
| b, p |
| q, g |
| C, O |
| I, l |


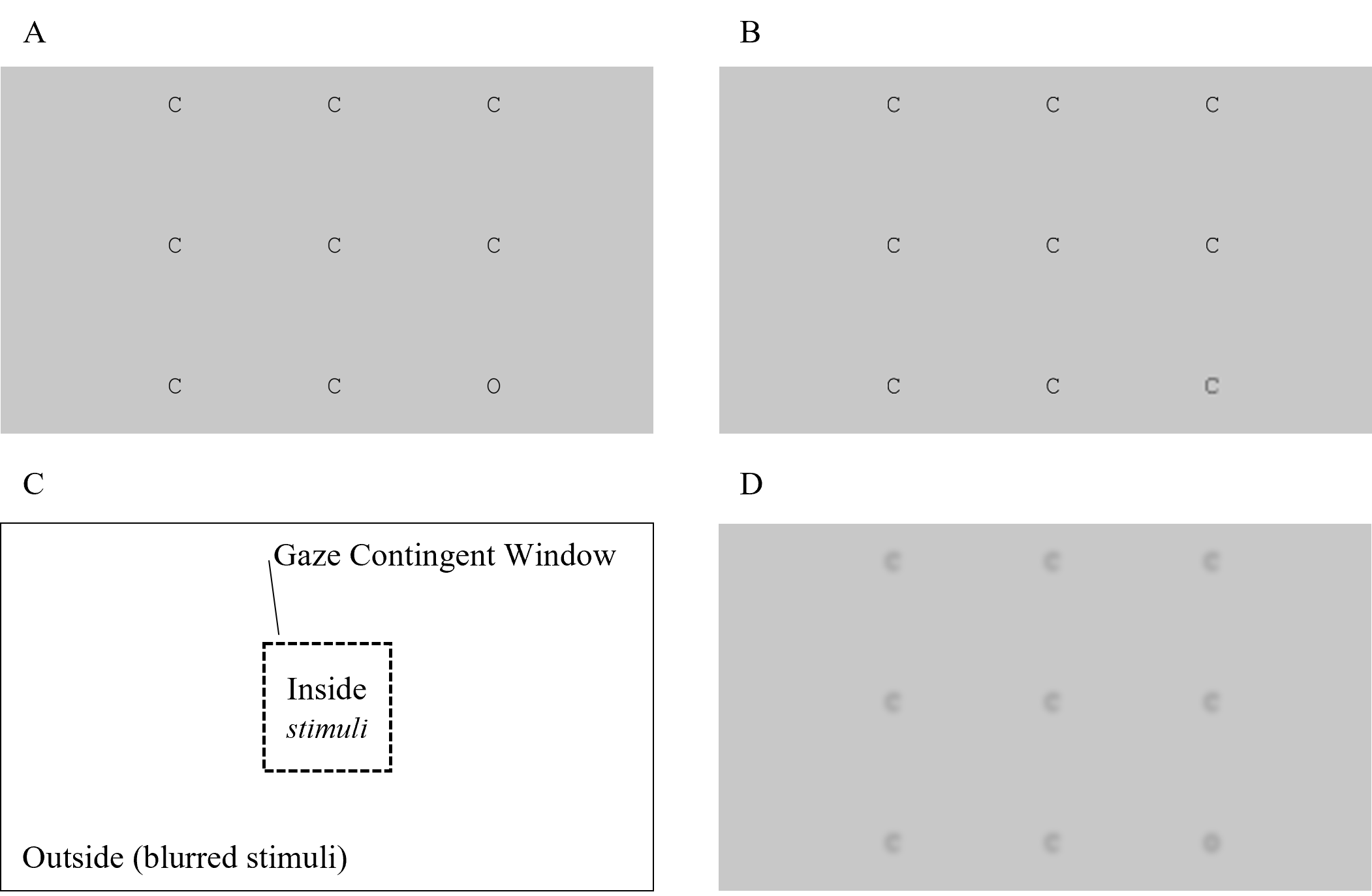


Fig S1. A) Stimuli image with the target character “O” placed at the bottom right. B) Background image. C) A virtual square window was set at the center of the participant’s fixated location. Stimuli were drawn inside the window, while mask images were drawn outside of the window. D) Example of the blurred stimuli (10-pixel standard deviation Gaussian blur). Since the visibility was reduced by blurring, we chose to use the mask image (B) rather than the blurred stimuli (D).





Fig S2. Average response time for English alphabet and Chinese characters visual search tasks

Appendix S4.

We investigated whether participants strategically placed their gazes between two nearby characters to inspect both characters simultaneously. This strategic behavior could potentially shorten the response times and create disturbances in our analysis. we calculated the fixation count per trial for each interest area on the stimuli (Table S4). The stimuli were divided into three areas of interest: 1) the gap region, where the partial region of each of the two Chinese characters could be rendered within the gaze-contingent window when the participant fixated on it (Fig S3, indicated in black); 2) the word region, where only one Chinese character could be rendered within the window (Fig S3, indicated in orange); and 3) the null region, where no character could be rendered within the window (Fig S3, indicated in blue). We calculated the representative average fixation count per participant (1,080 trials were averaged per participant) and then determined the mean and standard deviation, as shown in Table S4. There were very few fixations on the gap region, indicating that the participants did not place their gazes between the two adjacent characters to see both simultaneously. Furthermore, participants barely located their gaze on the null region, which indicates that they did not assess the action–effect temporal discrepancy by waving their gaze in the null region in the high visibility condition.


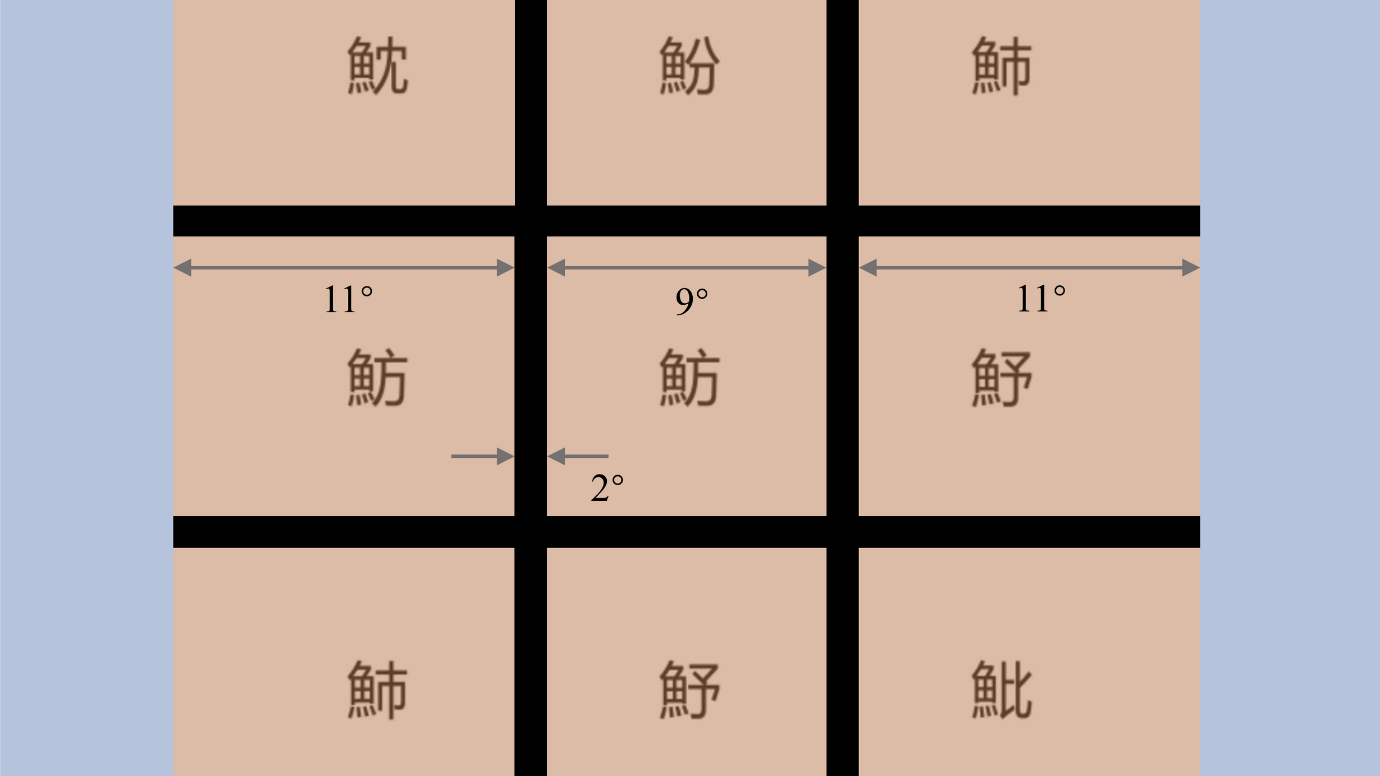


Fig S3. The stimuli were divided into three areas of interest: 1) Gap region, where partial sections of two Chinese characters can be rendered within the gaze-contingent window upon fixation (indicated in black); 2) Word region, where only one character can be rendered within the window (indicated in orange); and 3) Null region, where none of the characters can be rendered within the window (indicated in blue).

Table S4. Fixation count per trial for each region. Means and standard deviations were computed among the participants. There were very few fixations on regions other than the word region, indicating that task-unrelated behavior rarely occurred.

| Fixation count per trial | Gap region | Word region | Null region |
| --- | --- | --- | --- |
| Mean | 0.29 | 22.76 | 0.01 |
| Standard deviation | 0.15 | 5.74 | 0.01 |
